# Canine Placenta Histological Findings and Microvascular Density: The Histological Basis of a Negative Neonatal Outcome?

**DOI:** 10.3390/ani11051418

**Published:** 2021-05-15

**Authors:** Giuseppe Sarli, Carolina Castagnetti, Carlo Bianco, Giulia Ballotta, Giorgia Tura, Martina Caporaletti, Marco Cunto, Giancarlo Avallone, Cinzia Benazzi, Fabio Ostanello, Daniele Zambelli

**Affiliations:** 1 Department of Veterinary Medical Sciences, University of Bologna, Via Tolara di Sopra 50, Ozzano dell’Emilia, 40064 Bologna, Italy; giuseppe.sarli@unibo.it (G.S.); carolina.castagnetti@unibo.it (C.C.); carlo_blanc@yahoo.it (C.B.); giulia.ballotta2@unibo.it (G.B.); giorgia.tura3@unibo.it (G.T.); giancarlo.avallone@unibo.it (G.A.); cinzia.benazzi@unibo.it (C.B.); fabio.ostanello@unibo.it (F.O.); daniele.zambelli@unibo.it (D.Z.); 2 Health Science and Technologies Interdepartmental Center for Industrial Research (HST-ICIR), University of Bologna, Via Tolara di Sopra 41/E, Ozzano dell’Emilia, 40064 Bologna, Italy; 3 Clinica Veterinaria Tyrus, Via Aldo Bartocci 1/g, 05100 Terni, Italy; martinacaporaletti@gmail.com

**Keywords:** dog, placenta, histology, necrosis, capillary density, litter size

## Abstract

**Simple Summary:**

Accurate examination of the placenta, mainly by gross inspection rather than by histology, is common in clinical practice in some species, for example, in horses, but not carried out routinely in dogs. Placenta alterations in the mare can indicate malfunction, and data exist that intrauterine fetal nutrition influences both perinatal health, but also performances in adult life. Often placenta lesions are not easily appreciable on macroscopical examination, and histology is the best way to assess damage associated with sick newborns. The results of this paper underline the association between placenta injuries and outcome conditions in puppies and confirm, also in dogs, histological placenta investigation as a useful adjunctive tool in the evaluation of the newborn’s health and prognosis.

**Abstract:**

Placenta is essential for the development of the fetus, and its impaired function can lead to a negative outcome (i.e., neonatal mortality). In dogs, investigations on placenta histology and neonatal outcome in healthy bitches are lacking, and a contribution is provided in this study to emphasize the use of placenta histology in practice. Fifty-one placentas from 11 litters were collected during cesarean section, classified according to the litter size (large (L) or small (S)) and the outcome, this latter as healthy (Group 1) or dead within 7 days (Group 2). The placenta/puppy weight ratio (PPR) was calculated, and specimens were formalin-fixed and paraffin-wax embedded, and on the resulting histological slides, capillary density (CD) was quantified. Among necrosis, calcification, and intravascular leucocytes, only the presence of multifocal-confluent necrosis (significantly more frequent in Group 2) was associated with a higher risk of death within 7 days (odds ratio = 30.7). Mixed logistic regression ruled out the effect on death both of a bitch and cesarean type (programmed vs. emergency). PPR and CD values were associated with litter size; large litters had lower PPR (*p* < 0.01) and higher CD (*p* < 0.05) than small litters. The relationship between PPR and CD was negative and significant (*p* < 0.01). Necrosis was a frequent finding in canine placentas, but only when multifocal-confluent was it associated with a poor outcome. The litter size influenced PPR (lower in L) and CD (higher in L), and this is likely due to the plasticity of placenta adaptation.

## 1. Introduction

The formation of the placenta is essential for the correct development of the fetus and for a normal pregnancy. In humans, proliferation, differentiation, and cell death are the driving forces of placentation, determining the fate of the trophoblast cells [1]. Abnormalities at any stage of development, due to impaired proliferation, differentiation, or cell death, can lead to improper or pathological placental function and consequent complications related to pregnancy [1]. Focusing on veterinary medicine, the examination of the placenta is considered part of the obstetrical procedures in mares [2], and the proportion of sick foals is greater when placental abnormalities are observed [3,4]. In addition, the influence of the intrauterine life has been demonstrated as a determining factor of race performance in adult horses [5]. Different from the placenta in the horse, which is classified as of diffuse epitheliochorial nature, the canine placenta is zonary, lamellar, and endotheliochorial. The interface zone is the labyrinth composed of trophoblastic lamellae, in which cytotrophoblasts and syncytiotrophoblasts line the trophoblastic mesenchyme and cover the maternal vessels [6,7,8]. Canine syncytiotrophoblasts and cytotrophoblasts invade the maternal endothelium and endometrial glands early in gestation. These cells form “cuffs” around the maternal blood vessels and form the placental labyrinth [9,10]. Normal canine trophoblast invasion during implantation is relatively less invasive compared to other deciduate placental species (e.g., primates and rodents) [11]. Except for the marginal hematoma that forms in mid-gestation [9,10], canine trophoblasts do not come into direct contact with maternal blood [11]. Syncytiotrophoblasts are cells with up to ten centrally located nuclei and indistinct cell borders. Cytotrophoblasts are mononucleated, large cells that envelope maternal vessels, representing the interface between dam and fetus. The extracellular compartment of placental lamellae is inconspicuous [12].

The labyrinth is functionally organized in lobuli. Maternal stem arteries define the central axis of a lobule, while fetal stem arteries and veins occur in pairs and delimit the periphery of the lobule. Wherever these fetal vessels are sheathed by loose connective tissue, the lobule periphery becomes distinctly evident, as it happens in mink placentas. Arterioles branching from fetal and maternal stem arteries supply the respective capillary networks of the labyrinth and are drained by fetal and maternal venules [13].

Placental efficiency could be changed by alterations in the surface area for exchange, the thickness of the barrier between the maternal and fetal circulations, and/or in the density and architectural arrangements of the fetal and maternal vasculature within the placenta [14]. The natural intra-species variation in placental efficiency may be related to differences in the placental vasculature. In pigs and sheep, the breed differences in placental efficiency are associated with changes in capillary density with higher values in the more prolific breeds with smaller individual placentas and fetuses but higher fetal to placental weight ratios [14].

Data on the canine species aimed to investigate the clinico-pathological correlations between placenta histology and neonatal outcome in healthy bitches are still lacking. Few investigations describe gross and histologic features of canine placenta pathology, and case reports of reproductive disorders in experimental settings are sporadic [12,15,16,17,18]. Two recent papers on toy- and small-sized canine breeds focus mainly on macroscopic features and vessel area related to puppy weight [19] and on the description of histological findings in term placentas of healthy puppies [20].

The present investigation is aimed to correlate the histological changes in canine term placentas with neonatal outcomes and litter size.

## 2. Materials and Methods

Dogs submitted to cesarean section as elective surgery or undergoing emergency cesarean section at the Reproduction Unit of the Small Animal Clinical Service of the Department of Veterinary Medical Sciences—University of Bologna, Italy in the period November 2015–November 2018 were enrolled in this study following owners’ consent. All bitches included in the study were healthy, regularly vaccinated, and dewormed; the serum chemistry profiles performed before surgery were within normal limits. Five out of eleven bitches were pluriparous; none of them experienced any problem during previous pregnancies, while six were healthy primiparous bitches. For each case, the following data were collected: breed, weight, age, number of pregnancies (maternal data); APGAR score (assessed within five minutes from birth, according to Veronesi et al. [21]) body weight, placental weight, and outcome of each puppy (puppy data). Placental weight was assessed, measuring only the zonary placental region using a calibrated analytic scale in 1-g increments; the amnion and chorioallantois membranes and fluids were not considered. The placenta/puppy weight ratio expressed as a percentage (PPR) was calculated for each puppy. The whole placenta was fixed in 10% buffered formalin. Puppies were classified on the basis of the outcome: healthy (live born and healthy for 7 days after birth, Group 1), dead (live born but death occurring within 7 days after birth, Group 2). The litter size for each breed, in terms of the number of puppies, was compared with reference values [22]. If the litter size in the present study was greater than the reported mean value for the breed (litter size), it was defined as large (L); litters instead characterized by a lower number of puppies, compared with reference values, were defined small (S). As for the breeds not mentioned by Borges et al. [22], the litter size was defined large or small consistently with the live bodyweight of the breed and the criterion of affinity with other breeds belonging to the same morphological group according to the Fédération Cynologique Internationale (http://www.fci.be/en/nomenclature, accessed on 27 March 2021).

During the study period, data included in the dataset were from eleven parturitions, six of which scheduled and five emergency cesarean sections. Emergency cesarean sections were performed because of uterine inertia, no clinical sign of placental detachment was detected during clinical evaluation, and no fetus showed ultrasonographic signs of distress as reported by Lopate [23]. Fifty-one placentas from the eleven litters were included in the study (Table 1). A total of 43 placentas and puppy weights (13 from small litters and 30 from large litters) were acquired for the placental weight to puppy weight ratio (PPR) assessment.

The formalin-fixed placenta was trimmed to obtain an equatorial circumferential section, included in paraffin, and routinely processed for histology (hematoxylin-eosin stain and Alizarin stain of 4 μ thick sections).

### 2.1. Morphometry and Histopathology

For each case, 5 photographs, gif format, of random non-adjacent fields of the placental labyrinth were acquired (2088 × 1550 pixels, obj = 40×, 7.35 pixels µ, and field area 60,100 µ^2^) with a Leica microscope and Leica DFC320 DMLB camera (Leica, Wetzlar, Germany). Digital Image Analysis was carried out with ImageJ (http://imagej.nih.gov/ij/ accessed on 13 May 2017). Fetal capillaries of the labyrinth were counted using a point-counting technique with a superimposed grid (distance between the links = 22 µ; http://rsb.info.nih.gov/ij/plugins/grid.html accessed on 23 December 2017), the capillaries lying on the intersections of the meshes were counted (total 108 intersections). For each histological preparation, the mean value of capillary density (CD) was calculated.

### 2.2. Statistical Analysis

The distribution of the variables was assessed using the Shapiro–Wilk test. Pairwise comparison of normally distributed data (CD) was carried out with a Student’s *t*-tests, while the pairwise comparisons of non-normally distributed data (PPR) were performed with Mann–Whitney U tests. Spearman’s *rho* test was used to analyze the correlation between non-normally distributed data. Dichotomous variables were analyzed with a Chi-square test. Statistical significance was set as *p* < 0.05.

Finally, a mixed logistic regression model was performed to investigate the potential of cesarean typology (elective or emergency), litter size (large or small), placental necrotic changes (focal or multifocal-confluent), placental calcification pattern (focal vs. linear + sclerotic), CD, PPR, and APGAR score for puppies outcome (Groups 1 and 2). The puppies’ outcomes were the dependent variable, all other variables were the fixed factors, and the bitch was used as the random factor. Additionally, the Odds Ratio (OR) and its 95% Confidence Interval (95% CI) were estimated with a *p*-value < 0.05 considered to be statistically significant, quantifying the strength of the association between the puppies’ outcomes and fixed factors.

Statistical analysis was performed with Statistica 8 (Statsoft-Dell, TX, USA) and SPSS 26.0.0 (SPSS Inc., Chicago, IL, USA).

## 3. Results

### 3.1. Clinical Data and Placenta/Puppy Ratio

Histology was carried out on 51 placentas: 43 from healthy puppies (Group 1) and 8 placentas from puppies with a negative outcome (Group 2).

**Figure 1:**
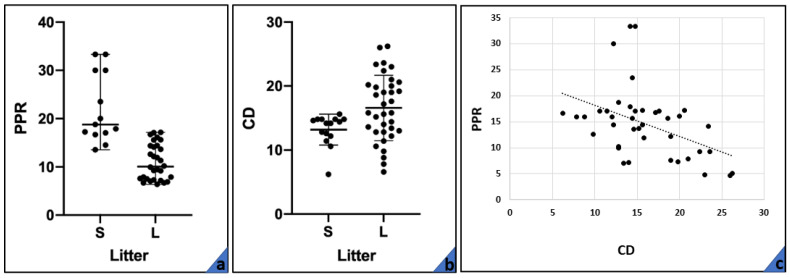
(**a**) Median and quartile range of PPR in small (S) and large (L) litters. (**b**) Mean and SD of CD in small (S) and large (L) litters. (**c**) Relationship between PPR and CD.

No differences in the PPR were detected between outcome Groups (Mann–Whitney U: 101; *p* = 0.42). The comparison of PPR between large and small litters was statistically significant (Mann–Whitney U: 39; *p* < 0.05): in large litters (*n* = 30), median PPR was 10.06%, with a range of 7.24–17.16, while in small litters (*n* = 13), median PPR was 18.78%, with a range of 13.55–33.33 (Figure 1a).

The median value of the APGAR score was 8 and 7 respectively for puppies belonging to Group 1 and Group 2. The main cause of death of the seven puppies belonging to Group 2 was Fading Puppy Syndrome (seven out of eight puppies) due to low birth weight and failure to thrive; one puppy died 2 h from birth due to unknown causes. Infectious causes were excluded on the basis of the clinical evaluation and parents’ history.

### 3.2. Histopathology and Morphometry

**Figure 2:**
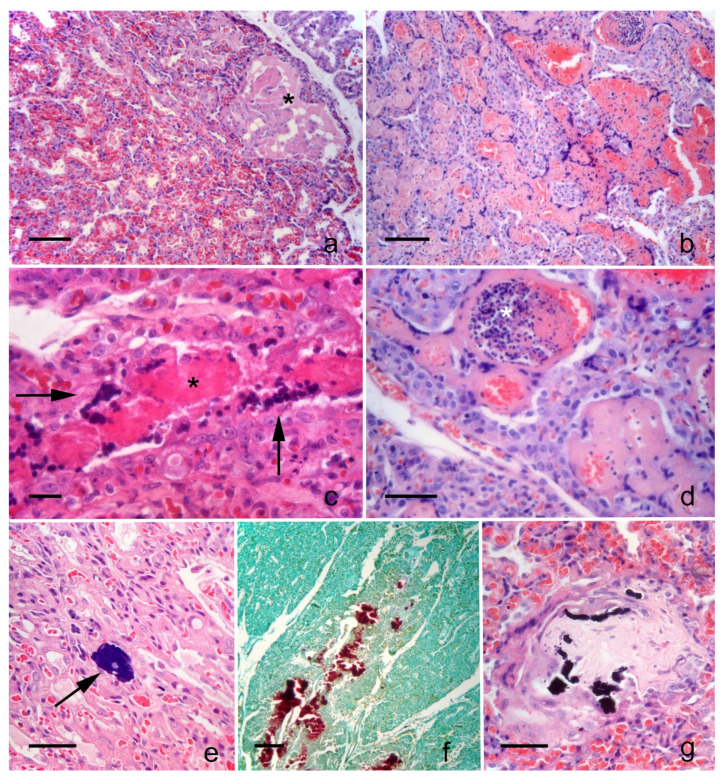
Placenta histological findings: (**a**) puppy of Group 1: focal necrosis of lamellae (asterisk); bar: 100 µm. (**b**) puppy of Group 2: large area of necrosis and hemorrhage. Bar 100 µm. (**c**) puppy of Group 2: highlights of vessel thrombosis (asterisk) in a necrotic lamella surrounded by a single layer of karyorrhectic trophoblasts (arrow); bar: 20 µm. (**d**) puppy of Group 2: necrosis of maternal vessels and intravascular karyorrhectic debris (asterisk); bar 50 µm. (**e**) puppy of Group 1: example of microfocal calcification (arrow) within a trophoblastic lamella; bar: 50 µm. (**f**) puppy of Group 2: linear pattern of mineralization, stained in red; bar: 200 µm. (**g**) puppy of Group 2: scattered, fragmented basophilic granular material embedded in mature collagen; bar: 50 µm. (**a**–**e** and **g**): hematoxylin-eosin stain; f: alizarin red stain for calcium.

The placental development, as determined by histological examination, was consistent with the gestational age. The most frequent lesions were degeneration and necrosis with focal, i.e., necrosis of one to three neighbor lamellae (Figure 2a) or multifocal-confluent, i.e., large area of necrosis associated or not to hemorrhage (Figure 2b) distribution. Areas with coagulative necrosis were well-demarcated, maintaining the outline of the lamellae (Figure 2a). Initial lesions evidenced vascular thrombosis and coagulative necrosis as shrunken and homogeneously eosinophilic syncytiotrophoblasts with loss of cellular details and chromatin pyknosis or karyorrhexis (Figure 2c). More severely affected areas were evidenced from cell swelling to coagulative necrosis in cytotrophoblasts. Other changes were scattered extravasated erythrocytes, and the maternal capillaries expanded with fibrin entrapping erythrocytes and a minimal amount of basophilic nuclear dust (karyorrhectic debris).

To compare degeneration/necrosis among the two outcome groups, the variable was considered dichotomous (absent or present) and the presence associated with the extension of the lesion (focal, multifocal, or multifocal confluent). Puppies of Group 1 showed degenerative-necrotic lesions in 18 out of 43 (41.9%) examined placentas; in 13/43 cases, the lesions were focal and multifocal-confluent in the remaining 5/43 cases. Puppies of Group 2 showed degenerative to necrotic lesions in 7/8 examined placentas (87.5%), 2/8 of which were focal and 5/8 multifocal-confluent. Overall, the proportion of placentas with necrotic lesions is significantly higher (Chi-square: 3.94; *p* = 0.047) in Group 2 (7/8; 87.5%) than in Group 1 (18/43; 41.9%). The Chi-square test showed that there was a significant difference in the proportion of the extension of the necrotic lesion between the two Groups (Chi-square: 11.72; *p* = 0.003). The multifocal distribution of necrosis and degeneration of placental labyrinth was more frequent in Group 2 (5/8; 62.5%) than in Group 1 (5/43; 11.6%) (Table 2).

In some areas with necrosis or degeneration, the presence of intravascular leukocytes in the lumina of maternal blood vessels was evident (Figure 2d), and in the surrounding trophoblasts, chromatin condensation (pyknosis) and a moderate cytoplasmic clear swelling were apparent. Intravascular leukocytes were present in 7 out of 43 placentas of Group 1 and in 1 out of 8 placentas of Group 2 (Table 2), and no statistically significant difference among groups was identified (Chi-square: 0.07; *p* > 0.05). Inflammatory cells were always associated with necrosis or degeneration of the placenta.

Another frequent finding was calcification that showed to have three patterns: (1) focal, (2) linear and extensive, (3) mineralization of sclerotic areas (Table 2). The first pattern was characterized by the presence of rare radially arranged foci of basophilic granular to slender needle-like material surrounded by syncytiotrophoblast and cytotrophoblast cells (Figure 2e). The second calcification pattern was linear and extensive, originating from the fetal side of the labyrinth and surrounded by trophoblast cells degenerated or palisading, with the presence of scant amounts of amorphous eosinophilic necrotic debris containing calcium, disclosed with alizarin red stain (Figure 2f). The third pattern of calcification was characterized by scattered fragments of basophilic granular material (mineralization) embedded in mature collagen-containing rare plump to stellated fibroblasts, delineated by a rim of trophoblastic cells (Figure 2g). Focal calcifications were detected within the labyrinth of 24 out of 43 puppies of Group 1 and 2 out of 8 of Group 2, but the difference in proportion was not significant (*p* > 0.05). Linear and sclerotic foci of calcifications of the trophoblastic mesenchyme (second and third mineralization patterns) were simultaneously present in none (0%) placentas of Group 1 and in 4 (50%) of Group 2 (Chi-square: 23.36; *p* < 0.05) (Table 2).

Among the additional histological findings, thrombosis of maternal arteries in one placenta of Group 2 and hematomas (similar to marginal hematophagous zone) was found in two subjects of Group 1 and in two subjects of Group 2.

The comparison of the capillary density (CD) between the outcome of Group 1 vs. Group 2 did not reveal any statistical differences (Student’s *t*-test: 0.31; *p* = 0.75). Placentas from small litters (*n* = 16) had a CD (mean ± sd) of 13.02 ± 2.41, while those obtained from large litters (*n* = 35) had a density of 16.58 ± 5.11 (Figure 1b). The difference was significant (Student’ *t*-test: 2.43; *p* < 0.05). The correlation between CD and PPR was negative, moderate, and significant (Spearman’s *rho*: −0.41631, *p* < 0.05) (Figure 1c).

Mixed logistic regression analysis (Table 3) showed an association between negative outcome (Group 2) and presence of focal necrosis (OR: 30.682; 95% CI: 1.805–1032.549; *p* = 0.049).

## 4. Discussion

As in human medicine, in veterinary medicine as well the placental histopathological examination may provide clinically useful data. In the present study, the histological lesions of canine term placentas are described, characterized, and quantified and their association with puppies’ outcome tested with the aim to consider them as background or incidental findings.

Focal or multifocal degeneration and necrosis were commonly observed as reported in human medicine [24] and more recently in a paper on term placentas of healthy miniature and toy and middle-sized dogs [20]. The architecture of the degenerative and necrotic lesions suggests that hypoxia was the most likely cause. Necrosis could be defined as placental infarctions because initial lesions appeared associated with vascular thrombosis. The timing of these lesions was hyperacute (moderate degenerative and necrotic changes mainly of syncytiotrophoblastic cells) or subacute (coagulative necrosis of both trophoblast cell types). This might reflect the duration of the ischemia. This evidence suggests that cytotrophoblast cells, which are believed to have histiocytic features [12], are putatively more resistant to hypoxia/anoxia, especially considering that they outline the stagnant blood lacunae of marginal placental hematoma. Assuming these lesions to be influenced by dystocia, we tested an effect of the cesarean type (elective vs. emergency) on death within 7 days after birth. Mixed logistic regression did not reveal different risks of death in puppies born by the two types of cesarean (OR = 4.7; *p* = 0.651), and this let us exclude the multifocal-confluent necrosis as an effect strictly linked to maternal dystocia of uterine inertia in the caseload used. The presence of subacute necrotic or their progression (chronic calcified) lesions emphasizes the genesis along with pregnancy and is not strictly associated with delivery.

Considering the results of statistical analysis among Groups, the focal or multifocal-confluent distribution patterns of placental degeneration and necrosis can have different influences on outcomes. If the lesion is focal, it has no consequences on the fetal and newborn outcome due to the efficiency of the residual placental tissue to compensate [25], and this feature is considered a normal finding in term canine placenta [20]. In the present study, the multifocal placental infarction was significantly more frequent in Group 2 and, in logistic regression, the only parameter associated with an increased probability of death within 7 days (OR = 30.7; *p* = 0.049). Consequently, the extent of the lesion may have influenced the outcome. The detection of focal degeneration and necrosis should be considered a background finding, as stated recently also by Tesi et al. [20], while the presence of multifocal distribution, if in conjunction with stem arteries thrombosis, should be deemed an important placental lesion affecting neonatal morbidity. The increased number of intravascular leukocytes does not seem to be a primary lesion but is most likely elicited by degenerative and necrotic changes.

Placental coagulative necrosis with various severity of placentitis was related to transmissible agents in dogs: *Brucella* spp., *Leptospira* spp., *Streptococcus canis*, *Canine herpesvirus*, *Neospora caninum*, *Toxoplasma gondii*, and *Leishmania* spp. [15,16,17,18,26], but evidence exists that placental necrosis is the consequence of a pure hemodynamic event: it was the main lesion in an experimental study in pregnant female dogs used as a model for human pre-eclampsia in which the lesion was caused only by prolonged ischemia [27] or in the placentas of healthy dogs with pharmacologic interruption of pregnancy [12].

Mineralization (calcification) was detected in three different patterns: the first can be defined microfocal and physiological; it occurs in the placenta of various species and can be interpreted as a background finding. Mineralization occurs because fetal blood is relatively more acid than maternal blood [28]. It should be stressed that this finding has never been associated with degeneration, necrosis, and/or inflammation. The second pattern of mineralization, linear and extensive, was always accompanied by degeneration/necrosis and was always found only in individuals of Group 2. This second pattern of calcification is consistent with dystrophic mineralization. The third pattern of mineralization is characterized by multiple foci of calcification in sclerotic areas that may represent a late stage of fibrotic evolution of a larger linear calcification because its coexistent with foci of extensive linear mineralization.

The presence of foci of hematophagous zones should be considered a background finding without pathological relevance, as it is often observed in the canine placenta [8].

This preliminary result shows that despite the median APGAR score of both groups of puppies being high (i.e., >7 according to Veronesi et al. [21]), there may be placental disorders that could lead, at least in our study, to death in the first week of life. In fact, the APGAR score evaluation allows a prompt identification of critical puppies at birth, which could be considered at risk of death during the first week of life and therefore require different degrees of medical assistance, but there is no guarantee that all those puppies showing higher APGAR scores will automatically survive [29].

Literature on the PPR has few examples in the dog to the best of our knowledge. One trial on Beagle dogs focused on the weight and gross morphometrical measures of placenta and puppies are available [30], but unfortunately, the litter size was not indicated, and pooled data were presented. In a recent paper, the PPR did not provide an association with the size of the dam, comparing toy- and middle-sized breed [19]. In the dog, we tried to compare PPR data between healthy and dead puppies, but both univariate and mixed logistic regression did not reveal any influence of PPR on neonatal outcome. In the Thoroughbred mare, instead, the normal weight of fetal membranes is 4.4–7.7 kg (or 11% of the foal’s weight at birth) [31]. If the membranes are significantly lighter, it implies that their surface area was smaller than normal, thus providing suboptimal nutritional support to the growing fetus. Membranes heavier than normal may be caused by edema and/or placentitis [32]. Further data have to be acquired in the dog to ascertain or not the meaning of the placental weight known for the foal.

The capillaries play a crucial role in the transport of oxygen and nutrients, and in the present study, perfusion was estimated in terms of capillary density, but no quantitative differences in microvascular density of placenta with the outcomes of puppies were noticeable. On the other hand, a “litter size effect” was evident on capillary density and PPR. Litter size displayed a moderate inverse correlation with capillary density, suggesting that a small placenta (in the case of large litters) gives rise to the exchange performance per unit area by increasing the capillarization. A similar result, albeit comparing different datasets, was reported in a recent paper from Tesi et al. [19], where the percentage of the area occupied by vessels (vascular index) had a negative correlation with puppy and placental weight. Even if microvascular density does not seem to influence puppy outcome, the result of this investigation suggests that the size of the litter modulates placental microvascular density in dogs.

Adopting a comparative point of view, the placental microvascular density is modulated in polytocous species of veterinary interest (porcine and ovine), and it is also known that the capillary density is greater in breeds with higher prolificacy, characterized by smaller placentas and increased fetoplacental ratio [14]. In light of these findings, the present study has putatively identified the same adaptation of increased capillary density in smaller placentas obtained from large litters. Angiogenesis, quantified by histological point count technique as the number of capillaries in trophoblastic lamellae (exchange area), confirms that the placenta is a plastic organ, adaptable to exchanging needs. The exchange performance is very likely to be higher per unit area in the placentas from large litters. In human medicine, the decrease in capillary density in placental villi was observed in pregnancies at an advanced maternal age, in pre-eclampsia, and in diabetes. The active or passive smoking and maternal anemia were instead related to an increase in capillaries (adaptive response to preplacental hypoxia) [33,34,35]. In the cases included in the present study, we had young adult bitches, of 4.4 ± 1.9 years of age, with clinical parameters within normal limits, and the influence of confounding factors (age, diseases) on placental microvascular density could be ruled out.

## 5. Conclusions

Since placenta histopathology provides insights about the fetal and neonatal outcome, in the present study, pathological changes of canine placenta were described and correlated to neonatal outcome. Overall, the CD and the PPR do not seem to have clinical or prognostic significance in the canine patient, whilst the placental histopathology can be informative in regards to the prognosis of the puppies at seven days. In particular, the multifocal-confluent necrosis, as revealed by histology, was significantly associated with a poor outcome for the puppies within seven days from birth. Because multifocal necrosis was the main finding associated with poor outcomes, it would be interesting to detect any alteration of placenta perfusion with advanced diagnostic imaging, reported as safe for use in pregnant mare and bitch [36,37], to have data to predict puppies outcome or plan intensive care at birth.

## Figures and Tables

**Table 1:** Clinical data collected for each litter and litter size (S: small; L: large).

Litter	Breed	Weight (Kg)	Age (Years)	No. of Pregnancy	Number of Puppies			Examined Placenta ^1^	Litter Size	Breed Mean Litter Size
					Total	Group 1	Group 2			
1	Shetland Sheepdog	6.5	4	1	3	3	0	3	S	3.9 ^a^
2	Boxer	35.8	-	-	11	9	2	11	L	6.6 ^a^
3	Pyrenean Mountain Dog	46.0	5	2	5 ^c^	1	2	3	S	7.0 ^b^
4	Maremma sheepdog	-	7	1	5	5	0	5	S	7.0 ^b^
5	Chihuahua	1.8	4	1	1	1	0	1	S	3.2 ^a^
6	Boston Terrier	8.3	1	1	3	3	0	3	S	4.1 ^a^
7	Mongrel	4.2	3	1	1	1	0	1	S	3.0 ^b^
8	Labrador	51.0	7	2	12 ^d^	3	-	3	L	6.9 ^a^
9	French Buldog	11.8	3	2	8 ^e^	4	3	7	L	4.7 ^a^
10	French Bullgog	10	4	2	7	6	1	7	L	4.7 ^a^
11	Bull Terrier	26	6	1	7	7	0	7	L	5.5 ^a^

^a^ Litter numerosity compared with reference values described by Borges et al. [22]; ^b^ Litter size as estimated on the basis of life body weight of the breed and criterion of affinity with other breeds belonging to the same morphological group according to the Fédération Cynologique Internationale (http://www.fci.be/en/nomenclature, accessed on 27 March 2021); S: small; L: large. Number of puppies born dead not included in the caseload: ^c^ 2 puppies; ^d^ 4 puppies; ^e^ 1 puppy; ^1^ the weight was available and the PPR calculated only for 43 out of the 50 placentas.

**Table 2:** Synopsis of major histopathological findings in placenta within outcome Groups.

Major Histopathological Findings		Group 1 Positive/Examined (%)	Group 2 Positive/Examined (%)	*p*-Value
Necrosis	focal	13/43 (30.2)	2/8 (25.0)	<0.05
	multifocal-confluent	5/43 (11.6)	5/8 (62.5)	
	absent	25/43 (58.2)	1/8 (12.5)	
Intravascular leukocytes	present	7/43 (16.3)	1/8 (12.5)	>0.05
	absent	36/43 (83.7)	7/8 (87.5)	
Calcification	focal	24/43 (55.8)	2/8 (25.0)	<0.05
	linear and sclerotic	0/43 (0.0)	4/8 (50.0)	
	absent	19/43 (44.2)	2/8 (25.0)	

**Table 3:** Results of mixed logistic regression analysis.

Model Term	OR	95% Confidence Interval for OR	*p*-Value
Elective cesarean	4.682	0.001–11,425.246	0.651
Large litter size	0.976	0.002–1039.957	0.993
Necrosis absent or focal	30.682	1.805–1032.549	0.049
Calcification pattern absent	0.101	0.004–2.183	0.139
Capillary density lower than the median value (14.6)	2.162	0.112–47.055	0.609
PPR lower than the median value (13.6)	0.414	0.001–206.851	0.765
APGAR ≥ 7	0.659	0.045–8.914	0.748
Intercept	1.482	0.003–502.057	0.892

## Data Availability

Data generated or analyzed during this study are included in this published article. The raw datasets used and analyzed during the current study are available from the corresponding author on reasonable request.
